# Visible‐Light Photo‐Iniferter Polymerization of Molecularly Imprinted Polymers for Direct Integration with Nanotransducers

**DOI:** 10.1002/smtd.202401315

**Published:** 2025-01-24

**Authors:** Tiziano Di Giulio, Muhammad Ibrar Asif, Martina Corsi, Giuseppe Egidio De Benedetto, Cosimino Malitesta, Karsten Haupt, Giuseppe Barillaro, Carlo Gonzato, Elisabetta Mazzotta

**Affiliations:** ^1^ Laboratory of Analytical Chemistry Department of Biological and Environmental Sciences and Technologies (Di.S.Te.B.A.) University of Salento via Monteroni Lecce 73100 Italy; ^2^ Information Engineering Department University of Pisa via G. Caruso 16 Pisa 56122 Italy; ^3^ Laboratory of Analytical Mass Spectrometry Cultural Heritage Department University of Salento Via Monteroni Lecce 73100 Italy; ^4^ CNRS Enzyme and Cell Engineering Laboratory Université de Technologie de Compiègne Rue du Docteur Schweitzer CS 60319 Compiègne 60203 France; ^5^ Institut Universitaire de France Paris France

**Keywords:** molecularly imprinted polymers, nanosensors, optical sensors, photo‐iniferter polymerization, porous silicon

## Abstract

Molecularly Imprinted Polymers (MIPs) have gained prominence as synthetic receptors, combining simplicity of synthesis with robust molecular recognition akin to antibodies and enzymes. One of their main application areas is chemical sensing. However, direct integration of MIPs with nanostructured transducers, crucial for enhancing sensing capabilities and broadening MIPs sensing applications, remains limited. This limitation mainly arises from the need for precise control over the MIP features (such as thickness) during deposition on nanostructured transducers. This work explores the potential of depositing MIPs directly onto nanostructured transducers via controlled radical photopolymerization, focusing on nanoporous silica (PSiO_2_) with pore sizes of 40 nm and aspect ratio exceeding 100 as an interferometric optical nanotransducer. Leveraging the covalent attachment of a photo‐iniferter agent onto the PSiO_2_ surface, we achieved effective control over the polymerization process, resulting in the deposition of thin and uniform MIP layers on PSiO_2_. As a case study, we developed an MIP‐based PSiO_2_ optical sensor for propranolol, used as the template molecule, showcasing excellent linearity, a low detection limit, and efficacy in real matrices such as tap water. The results further demonstrate the sensor selectivity for the target molecule, along with its reusability and stability for at least 60 days.

## Graphical abstract

This study overcomes the challenges of integrating MIPs with nanostructured transducers for chemical sensing. It demonstrates precise MIP deposition on nanoporous silica (PSiO_2_) via controlled radical photopolymerization, achieving uniform thin films. Using propranolol as the template, the resulting MIP‐based PSiO_2_ optical sensor offers high sensitivity, selectivity, and stability, with successful application in real matrices like tap water and a 60‐day lifespan.

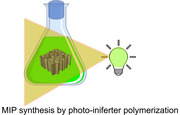

## 1 Introduction

Molecularly Imprinted Polymers (MIPs) are synthetic materials designed to mimic molecular recognition capabilities observed in natural biological systems, such as antibodies and enzymes.^[^ ^1^, ^2^, ^3^, ^4^, ^5^, ^6^ ^]^ In MIP synthesis, a polymer forms around a specific target molecule, which is subsequently removed. This process generates cavities within the polymer matrix that retain a molecular fingerprint, matching the shape, size, and functional group distribution of the original target molecule.^[^ ^1^, ^7^, ^8^, ^9^, ^10^, ^11^ ^]^ Over the last decades, MIPs have gained significant attention as synthetic receptors in sensing applications,^[^ ^7^, ^8^, ^12^, ^13^, ^14^, ^15^, ^16^, ^17^, ^18^, ^19^, ^20^ ^]^ thanks to their improved stability, cost‐effectiveness, and simple preparation compared to their biological counterpart.^[^ ^1^, ^12^, ^21^, ^22^, ^23^ ^]^ In sensing applications, the direct integration of MIPs with the transducer is beneficial as it affords ready‐to‐use devices without the need for additional steps to couple MIPs to the transducer surface. Further advantages in terms of increased exposed area, and, in turn, enhanced binding kinetics^[^ ^24^, ^25^ ^]^ come from MIPs scaling down to nanosize, which can be combined with the direct integration with the transducer in two ways, namely by depositing nanosized MIPs on planar transducers or MIP films on nanostructured transducers. In the first case, MIP nanomolding^[^ ^26^, ^27^, ^28^, ^29^ ^]^ and nanopatterning^[^ ^24^, ^30^ ^]^ are suitable techniques. Nanomolding involves etching sacrificial scaffolds, such as nanoporous alumina membranes and silica nanoparticles, to obtain MIPs in the form of nanofilaments, nanofibers, or nanophotonic crystals. On the other hand, nanopatterning requires additional nanolithographic steps, which increase the complexity of the fabrication process.

Only a few examples of one‐step strategies enabling direct integration of MIPs with nanotransducers have been reported so far, including electropolymerization,^[^ ^31^, ^32^, ^33^, ^34^ ^]^ dopamine self‐polymerization,^[^ ^31^, ^35^ ^]^ and the recently proposed vapor‐phase polymerization^[^ ^36^ ^]^ and highly localized polymerization.^[^ ^37^ ^]^

Electropolymerized MIPs have been conveniently obtained on nanostructured electrodes^[^ ^31^, ^32^ ^]^ leveraging the precise control over the polymerization process provided by the electrochemical synthesis. Nonetheless, their limitation to electroconductive materials restricts their broader application. MIPs based on polydopamine via self‐polymerization have also been increasingly used in sensing applications, exploiting the substrate‐independent polymerization mechanism^[^ ^38^, ^39^ ^]^ to facilitate their easy and rapid integration with any material.^[^ ^40^ ^]^ However, the limited control over polydopamine polymerization often results in the aggregation of dopamine micro‐/nanoparticles, leading to high surface roughness.^[^ ^41^, ^42^ ^]^ This compromises reliable polymer deposition, especially on nanostructured surfaces.

Recently, a vapor‐phase polymerization approach has been developed for the synthesis of a MIP for human hemoglobin directly integrated with nanostructured transducers.^[^ ^36^ ^]^ This approach enables the deposition of homogeneous MIP films with controlled thickness on nanostructured transducers at ambient temperature and pressure, without the need for any additional post‐deposition steps. Highly localized polymerization has been also reported for MIP direct integration on nanotransducers, consisting of the high confinement of the stimulus responsible for triggering the polymerization in close proximity to the transducer. In this way, very thin imprinted films have been deposited on quantum dots^[^ ^21^ ^]^ and gold nanoparticles (NPs).^[^ ^37^ ^]^ Highly localized polymerization can be also achieved by the covalent attachment of a chemical species capable of surface‐initiated radical polymerization to the transducer surface. While this strategy has been effective in controlling MIP thickness,^[^ ^43^ ^]^ it has not been applied to nanotransducers yet.

In the present work, we harness low‐power, visible light‐mediated photo‐iniferter polymerization for the direct deposition of MIPs on nanoporous silica (PSiO_2_), with a high aspect ratio (> 100) and columnar pores with a size ≈40 nm, serving as a nanostructured optical transducer.^[^ ^44^, ^45^, ^46^, ^47^ ^]^ A thin MIP film that conformably replicates the PSiO_2_ surface is achieved by attaching the photo‐iniferter agent to the PSiO_2_ surface. As a proof of concept, MIPs against propranolol and atenolol as model targets are synthesized, and the resulting sensor is used for the target detection in different matrices, demonstrating high selectivity, reproducibility, and stability.

## 2 Results and Discussion

**Figure 1:**
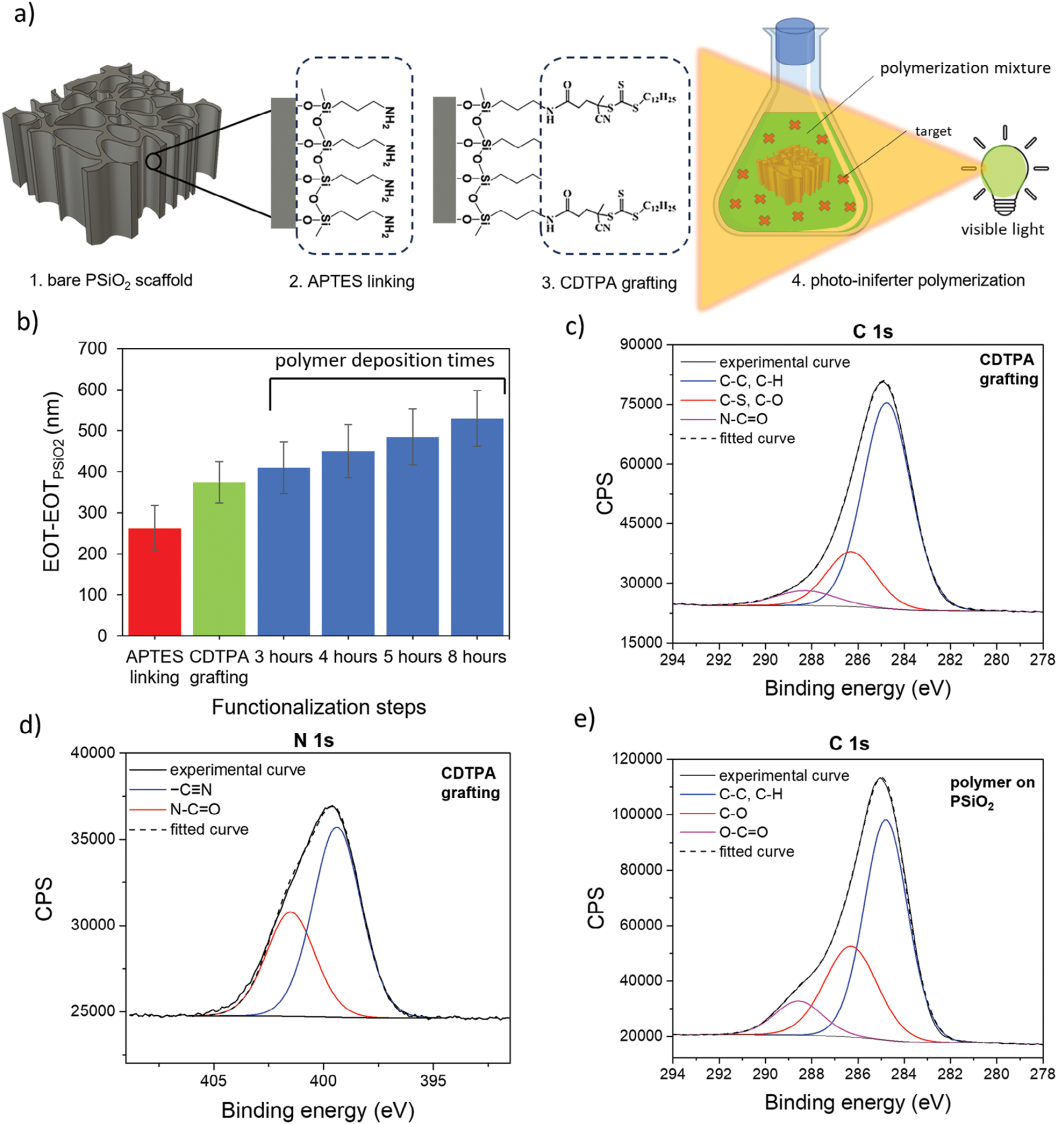
Photo‐iniferter polymerization on PSiO_2_ scaffold. a) Schematic representation of photo‐iniferter polymerization procedure, which includes the modification of the bare PSiO_2_ (1) with APTES (2), CDTPA grafting through a coupling reaction (3), photoiniferter polymerization (4) to obtain a poly(MAA‐co‐EGDMA) film. b) Effective optical thickness changes (EOT‐EOT_PSiO2_) recorded after each PSiO_2_ functionalization step, up to the polymer deposition for different polymerization times, namely 3, 4, 5, and 8 h (*n* = 3). EOT_PSiO2_ is the signal recorded for the bare PSiO_2_ scaffold. Data are presented as the mean of 3 independent samples (± s.d). High‐resolution C 1s (c) and N 1s (d) XPS spectra recorded after CDTPA grafting on PSiO_2_. Signals are fitted and corrected for charging. e) High‐resolution C1s spectrum recorded after polymer deposition on PSiO_2_. Signals are fitted and corrected for charging.

**Figure 2:**
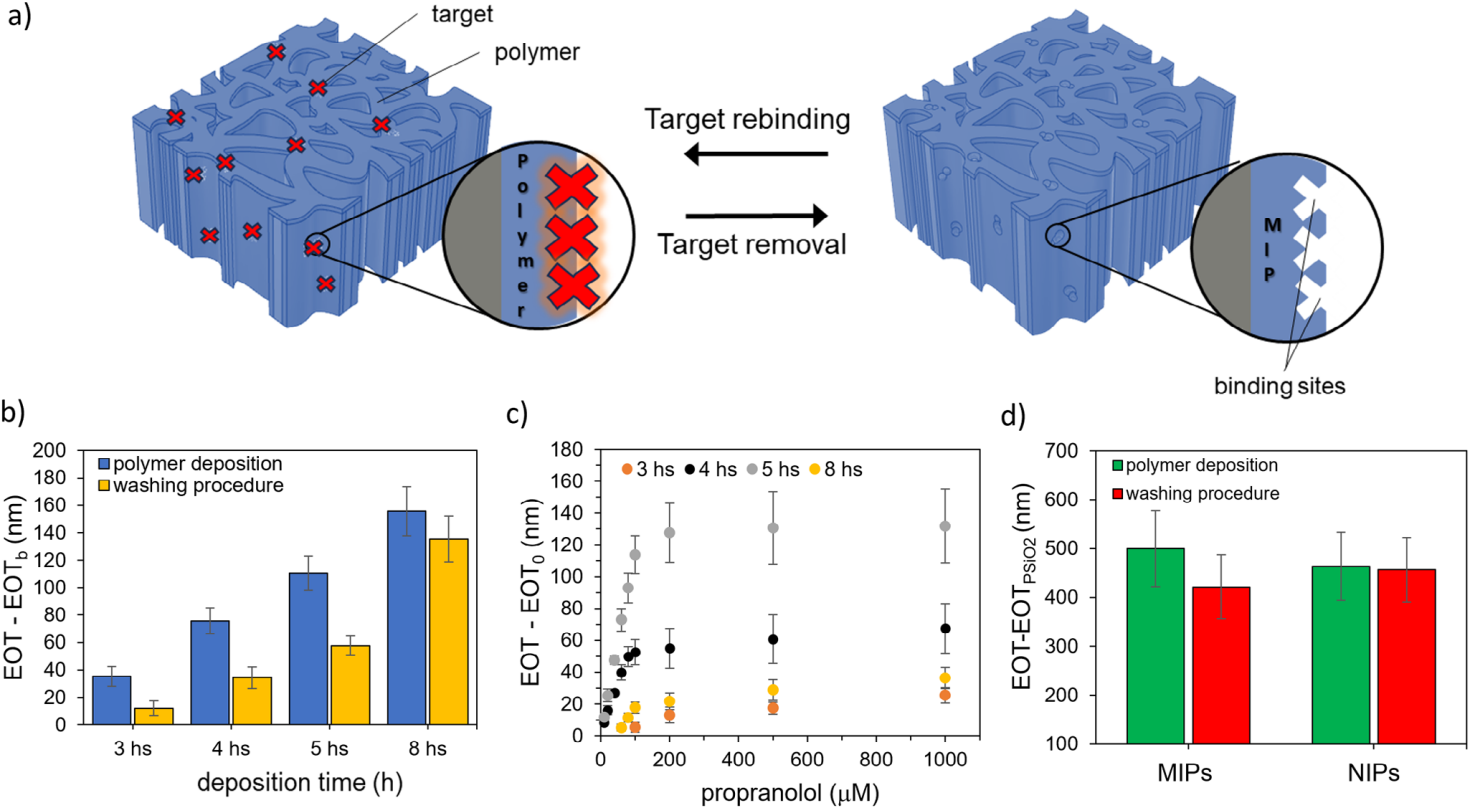
Molecularly imprinted polymers for propranolol on PSiO_2_‐based interferometer by photo‐iniferter‐polymerization. a) Schematic representation of the reversible interaction of propranolol (red crosses) with MIP on PSiO_2_ scaffold. b) Effective optical thickness changes (EOT‐EOT_b_) recorded before (blue bars) and after (yellow bars) the washing procedure on polymers obtained at different deposition times. EOT_b_ is the signal recorded before the polymer deposition (*n* = 3). c) Responses to propranolol concentrations recorded on MIPs obtained at different deposition times (*n* = 3). EOT_0_ is the signal recorded for a blank solution. d) Changes of effective optical thickness (EOT‐EOT_PSiO2_) as a consequence of the washing procedure on MIP and NIP (*n* = 3). The EOT value of the bare PSiO_2_ scaffold (EOT_PSiO2_) is used as a reference. Data are presented as the mean of 3 independent samples (± s.d).

**Figure 3:**
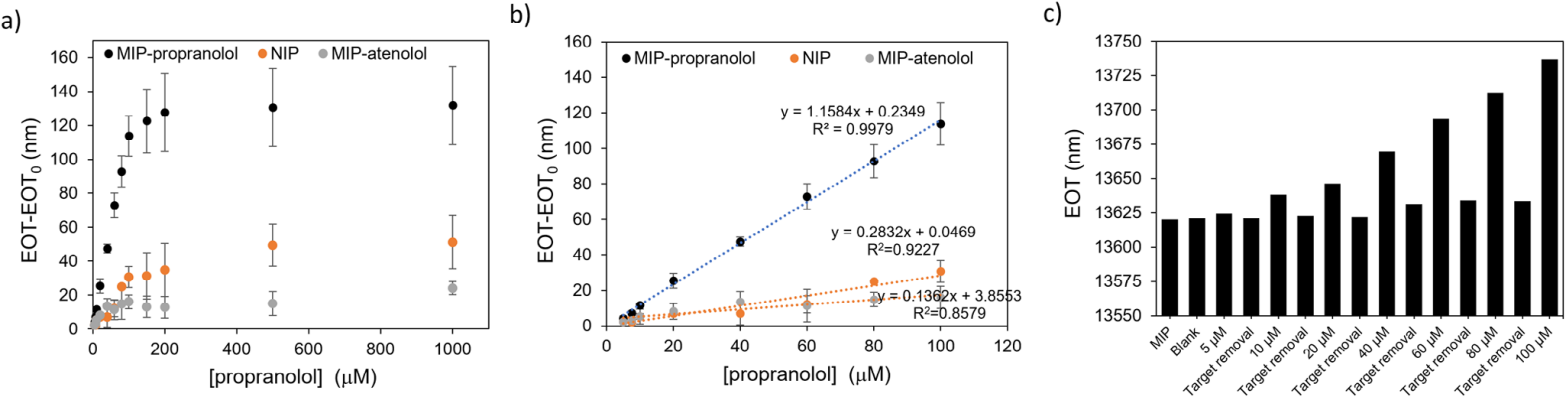
Evaluation of MIP‐PSiO_2_ performances toward propranolol by optical detection. a) Calibration curves (EOT‐EOT_0_ versus propranolol concentration) recorded using MIPs for propranolol (black dots), not imprinted polymers, NIPs (orange dots), and MIPs for atenolol (gray dots), testing propranolol solutions in acetonitrile, in the range 5–1000 µm (*n* = 3 samples). Data are presented as the mean of three independent samples (± s.d.). b) Calibration curves (EOT‐EOT_0_ versus propranolol concentration) recorded using MIPs for propranolol (black dots), not imprinted polymers, NIPs (orange dots), and MIPs for atenolol (gray dots), testing propranolol solutions in the range 5–100 µm. c) Monitoring EOT signal on a single MIP‐functionalized PSiO_2_ sample after incubation with propranolol solutions at different concentrations (5–100 µm) and sensor regeneration by the washing procedure.

The deposition of a MIP layer on PSiO_2_ scaffolds via photo‐iniferter polymerization is schematically illustrated in **Figure** **1**a. The first step involves silanization of the PSiO_2_ scaffold with APTES to anchor amino groups onto its surface (Figure 1a‐1). This step facilitates the subsequent coupling of the photo‐iniferter agent 4‐cyano‐4‐[(dodecylsulfanylthiocarbonyl) sulfanyl]pentanoic acid (CDTPA) (Figure 1a‐2). Upon exposure to visible light, CDTPA triggers surface‐initiated growth of the MIP layer, using methacrylic acid (MAA) and ethylene glycol dimethacrylate (EGDMA) as the functional monomer and crosslinker, respectively (Figure 1a‐3). Each step is monitored through UV–vis reflectance spectroscopy (Figure S1, Supporting Information) and by X‐ray photoelectron spectroscopy (XPS) to track the chemical modifications occurring at the surface of the PSiO_2_ scaffold.

Figure 1b summarizes the changes in the effective optical thickness (EOT) retrieved through reflectance spectroscopy after each functionalization step. A redshift in the reflectance spectrum (Figure S2, Supporting Information) indicates the successful anchoring of APTES,^[^ ^36^ ^]^ quantified by an increase in the EOT value. The EOT of the bare PSiO_2_ scaffold, namely EOT_PSiO2_, is used as the reference. The successful silanization is also consistent with XPS data, as evidenced by the appearance of C1s and N1s signals in the wide spectrum of PSiO_2_ (Figure S3a,b, Supporting Information). More specifically, fitting of C1s and N1s high‐definition spectra (Figure S4, Supporting Information) allows the identification of the different species. Besides the obvious presence of ‐NH_2_ groups at ≈399 eV, a component at 401 eV due to quaternary nitrogen suggests proton transfer from surface silanols to amino moieties^[^ ^48^ ^]^ (Figure S4a, Supporting Information). C1s spectrum (Figure S4b, Supporting Information) shows a dominant component at 284.8 eV related to the hydrocarbon chain of APTES silane and a component at higher binding energy (286.4 eV), ascribed to the contributions of C─N and C─O groups, related to amino groups and possible unreacted ethoxy groups, respectively.^[^ ^48^ ^]^

Upon CDTPA anchoring to the APTES‐modified PSiO_2_, a redshift of the reflectivity spectra is observed (Figure S2, Supporting Information), corroborated by the increase in the EOT value (Figure 1b). The successful coupling of CDTPA is also confirmed by XPS analysis. The survey scan (Figure S3c, Supporting Information), along with an increase in the intensity of the C 1s and N 1s regions, indicates the appearance of the S 2p signal, distinctive of CDTPA and related to the C─S and C═S groups. By fitting the C1s peak (Figure 1c), several components can be identified. Along with those at 284.8 and 286.3 eV, which reveal aliphatic and oxidized carbon species (i.e., C─S, C─N, and C═S), the appearance of a third component at 288.4 eV possibly relates to the amide group (N─C═O). At the same time, the successful grafting is also supported by the fitting of N1s high‐definition spectrum (Figure 1d), which shows the increase of the component at higher binding energies (401.5 eV), related to amide functional groups.^[^ ^49^, ^50^, ^51^ ^]^ After CDTPA grafting on PSiO_2_ scaffolds, the nitrogen content (evaluated by N/Si atomic ratio) also increases due to the cyano group (Figure S5a, Supporting Information), along with C/Si atomic ratio (Figure S5b, Supporting Information) increasing from 0.54 to 1.54.

CDTPA is a visible light‐sensitive photo‐iniferter, also serving as a chain transfer agent in reversible addition‐fragmentation chain transfer (RAFT) polymerization.^[^ ^52^ ^]^ When exposed to low‐intensity visible light (ranging from blue to green), a small fraction of CDTPA reversibly dissociates into a tertiary‐carbon radical, capable of initiating polymerization, and a dormant radical. At the same time, the remaining CDTPA supports the chain transfer, helping to achieve controlled conditions. MIP nanolayers within the nanostructured PSiO_2_ are thus grown via surface‐initiated polymerization with no further treatment to remove PSiO_2_, as it acts as a transducer for MIP sensing applications. Grafting a species capable of controlled polymerization as CDTPA to the nanostructured surface has two main advantages: first, it provides control over the polymer growth and adhesion within the nanopores of PSiO_2_; second, it prevents the growth of free oligo/polymer in the polymerization solution, thus avoiding the possible blocking of nanopores.

To assess the efficacy of the proposed functionalization protocol and to compare different polymerization times, preliminary tests on photo‐iniferter polymerization were carried out on flat Si samples. Contact angle (CA) measurements were used to monitor changes in surface hydrophobicity/hydrophilicity, resulting from polymer deposition and previous functionalization steps.^[^ ^53^ ^]^ The results are shown in Figure S6 (Supporting Information). The silanization increases the contact angle to 48.2±4.2°, compared to oxidized Si samples (37.1±2.2°), consistent with literature data.^[^ ^54^ ^]^ Upon CDTPA grafting, the contact angle further increases (66.4±5.6°), possibly due to the hydrophobic dodecyl end group of the photo‐iniferter agent. CDTPA‐grafted samples were then placed in a solution containing MAA and EGDMA as monomer and crosslinker, respectively, and exposed to the green light at 1.5 mW/cm^2^ to grow a poly(MAA‐co‐EGDMA) layer. This light intensity was selected to minimize the risk of photobleaching on CDTPA.^[^ ^55^ ^]^ Various deposition times were tested, ranging from 40 min to 8 h. A significant increase of CA is observed after 3 h of polymerization, (81.3±2.7°), possibly resulting from the aliphatic chains of poly(MAA‐co‐EGDMA) deposited on the samples. The 3‐h polymerization condition was thus selected as the starting condition for polymer deposition on PSiO_2_ scaffolds, along with a longer time.

CDTPA‐grafted PSiO_2_ scaffolds were exposed to the polymerization mixture and irradiated with green light, investigating different polymerization times, namely 3, 4, 5, and 8 h. Reflectance spectra acquired after polymer deposition for these times are shown in Figure S7 (Supporting Information). A significant red‐shift in the reflectance spectra is evident already after 3 h of polymerization, which further increases for longer times, suggesting successful polymer deposition within the pores of the PSiO_2_. EOT values consistently increase with extended polymerization (Figure 1b), indicating an increased polymer deposition within the nanopores.^[^ ^36^ ^]^

Polymers synthesized within PSiO_2_ scaffolds were then analyzed using XPS. The recorded high‐resolution C 1s peak (Figure 1e) consists of three different components assigned to ─C─C, ─C─H (284.8 eV), C─S, C═S, C─O (286.4 eV), and O─C═O (288.5 eV). The increase of intensity of the two components related to oxidized carbon, compared with the C 1s signal recorded after CDTPA grafting (Figure 1c), are related to poly(MAA‐co‐EGDMA), with ─C─O and O─C═O originating from both EGDMA and MAA.^[^ ^56^ ^]^ Interestingly, the same components are also detected in the C 1s spectrum recorded on a bulk poly(MAA‐co‐EGDMA) (Figure S8, Supporting Information), synthesized in solution (not on PSiO_2_ scaffolds) under identical conditions but with CDTPA in the polymerization mixture. Additionally, a consistent increase in the C/Si atomic ratio (Figure S5b, Supporting Information) is evident after polymerization, ascribable to the carbon backbone of the deposited polymer, as clearly seen also in the survey scan (Figure S3d, Supporting Information).

MIP deposition on PSiO_2_ scaffolds was then performed by adding propranolol to the polymerization mixture as a molecular target and testing different deposition times, namely 3, 4, 5, and 8 h (**Figure** **2**a). The washing procedure (described in Experimental Section) resulted in a blueshift of the reflectance spectrum and a corresponding reduction of the EOT value for all the polymerization times (Figure 2b), indicating the effective removal of the target analyte from the polymer film, a crucial step in MIP preparation. Notably, the extent of EOT decrease varied with the polymerization time, ranging from 70% for the MIP achieved after 3 h, 60% for 4 and 5 h, to 10% after 8 h. This trend aligns with the thicker polymer layer growing inside the PSiO_2_ scaffold as the polymerization time increases, progressively trapping the template molecule in the polymer bulk that cannot be easily removed by the washing step.

Figure S9a (Supporting Information) shows the typical morphology of a PSiO_2_ layer, featuring columnar nanopores with an average size of ≈40 nm. SEM top‐views of PSiO_2_ scaffolds coated with MIP after 5 h of polymerization (Figure S9b, Supporting Information) closely resemble the bare PSiO_2_ scaffold, with a similar average size and distribution of pores sizes (inset in Figure S9a,b, Supporting Information). These observations suggest that a uniform layer of polymer, only a few nanometers thick, is evenly deposited inside the pores. This aligns with the redshift observed in the reflectance spectrum after MIP deposition, and consequently, with the increase in the EOT value, compared to bare PSiO_2_ (Figure 1b; Figure S2, Supporting Information).

As a control, non‐imprinted polymers (NIPs) were also prepared on PSiO_2_ samples under the same conditions, though without a target compound in the polymerization mixture. Top‐view SEM images after 5 h of polymerization show that the morphology of NIP‐ (Figure S9c, Supporting Information) and MIP‐coated PSiO_2_ scaffolds are similar, with a pore reduction of 10 nm in diameter in both cases. Specifically, the variations in the estimated equivalent diameter for the MIP and NIP compared to the bare silica scaffold (PSiO_2_) are calculated as follows:

$\frac{d_{MIP}}{d_{PSiO2}}=\frac{d_{NIP}}{d_{PSiO2}}=0.75$, where d_PSiO2_, d_MIP_, and d_NIP_ correspond to the mean diameter values extracted from the SEM images of the bare porous silica scaffold and of the porous silica scaffolds with MIP and NIP, respectively (see insets of Figure S9, Supporting Information). The analyses thus demonstrate a 25% reduction in pore diameter after the MIP and NIP deposition.

Similarly to MIP, NIP synthesis was monitored by UV–vis spectroscopy and the EOT values were recorded after each functionalization step. Figure 2d shows the EOT values obtained for MIP and NIP after 5 h of polymerization, respectively. The change in the EOT values is similar for MIP and NIP, whereas the washing step does not result in any significant EOT variation for the NIP. This result further confirms the efficacy of the washing step in removing the target analyte from the MIP and provides indirect evidence of the polymer stability under the washing conditions tested.

Kinetic binding studies were conducted on MIP‐coated PSiO_2_ scaffolds using a 50 µm propranolol solution in acetonitrile, monitoring changes in EOT after incubation over a time span from 5 to 60 min. As shown in Figure S10 (Supporting Information), which refers to MIPs obtained from 5 h of polymerization, the signal increases over time up to 30 min, after which it stabilizes indicating possible saturation of the MIP binding sites. Therefore, an incubation time of 30 min was selected for subsequent binding studies.

The sensing performance of MIP‐functionalized PSiO_2_ scaffolds for propranolol detection was evaluated using propranolol solutions at different concentrations in the range 10–1000 µm. Figure 2c compares the calibration curves obtained for MIPs prepared on PSiO_2_ scaffolds for polymerization times ranging from 3 to 8 h. The calibration curves indicate that MIPs prepared at 4 and 5 h exhibit higher sensitivity compared to 3 and 8 h. Minimal or no signals were recorded for propranolol concentrations <100 µm on MIPs prepared at 3 and 8 h, respectively. This result may be attributed to the thin layer formed after only 3 h, which possibly lacks sufficient binding sites for specific recognition. Conversely, the MIP prepared for 8 h may have a thick and compact layer that hinders template removal, thus limiting its binding capacity. A comparison of the performances of MIPs prepared at 4 and 5 h reveals that the latter exhibits significantly higher responses across the whole concentration range and is therefore selected for further MIP characterization and testing.

An indirect estimation of MIP thickness was conducted using XPS analysis. XPS offers the possibility to estimate the thickness of layers or films deposited on surfaces by measuring changes in the intensity of photoelectron signals before and after deposition, under certain assumptions.^[^ ^57^, ^58^ ^]^ Here, the attenuation of the Si 2p signal after polymer deposition was utilized to estimate film thickness using the formula:^[^ ^48^, ^57^ ^]^

(1) $$I_{Si}^{polymer}/I_{Si}^{PSiO2}=exp^{\left(-t/\lambda_{Si}^{polymer}\right)}\;$$

where $I_{Si}^{PSiO2}$ and $I_{Si}^{polymer}$ represent the Si 2p intensity recorded before and after polymer deposition, respectively, *t* is the film thickness, and $\lambda_{Si}^{polymer}\;$ is the attenuation length, calculated on the basis of the experimental data^[^ ^57^ ^]^ (assuming a density for poly(MAA‐co‐EGDMA) equals 1.19 g cm^−3^,^[^ ^59^ ^]^ corresponding to 34 A. The estimated thickness for MIP prepared over 5 h is 3.7±0.6 nm. Although these estimates are derived from a simplified model that does not fully account for the nanostructured PSiO_2_ surface, they are consistent with previously reported data for similarly obtained MIPs.^[^ ^60^ ^]^

We further performed MIP‐ and NIP‐coated PSiO_2_ scaffold (obtained by 5 h of polymerization) characterization by Raman spectroscopy (Figure S11, Supporting Information). Raman spectra were recorded for the bare PSiO_2_ scaffold as well as for NIP‐ and MIP‐coated PSiO_2_ samples. Additionally, a bulk polymer was synthesized using the same polymerization method but with a solution containing the photo‐iniferter (at 1% of the total concentration of monomer and crosslinker) and then was used as a reference for spectral interpretation.

The Raman spectrum of the bare PSiO_2_ scaffold exhibited two major peaks: a prominent peak at ≈511 cm⁻¹ corresponding to Si─Si stretching and a broad peak ≈930 cm⁻¹ attributed to multi‐phonon scattering in the Si substrate.^[^ ^61^ ^]^ Less intense vibrational features were also observed at 230 and 300 cm⁻¹, corresponding to SiO_2_ tetrahedral scissoring and Si─O─Si bending modes, respectively, as previously reported in the literature.^[^ ^61^ ^]^

Raman spectroscopic analysis of the NIP‐ and MIP‐coated PSiO_2_ scaffolds revealed distinct vibrational peaks characteristic of the poly(MAA‐co‐EGDMA) copolymer, in line with the literature.^[^ ^62^, ^63^, ^64^, ^65^ ^]^ The most prominent bands were observed at 1725, 1450, and 600 cm^−^¹, along with additional peaks at 1640, 1405, 875, and 810 cm⁻¹. The strong band at 1725 cm⁻¹ is attributed to C = O stretching vibrations, indicative of the ester carbonyl groups present in both MAA and EGDMA units.^[^ ^63^, ^64^, ^65^ ^]^ This peak confirms successful polymerization and the formation of cross‐linked ester structures within the polymer matrix. The band at 1450 cm⁻¹ corresponds to CH₂ bending vibrations, characteristic of aliphatic chains, and suggests the presence of methylene bridges formed as part of the cross‐linked structure.^[^ ^62^, ^63^, ^64^ ^]^ The peak at ≈600 cm⁻¹ is assigned to C─C skeletal vibrations or bending modes involving substituted aliphatic groups, possibly associated with cross‐linking or secondary interactions within the polymer matrix.

The additional peak at 1405 cm⁻¹ is attributed to CH bending vibrations or deformation modes, which contribute to the vibrational profile of the hydrocarbon framework of the polymer. The peaks at 885 and 810 cm⁻¹ correspond to out‐of‐plane C─H bending and deformation associated with substituted alkenes, further supporting the formation of a copolymer architecture involving MAA and EGDMA units.^[^ ^62^, ^63^ ^]^ These signals were also detected in the bulk polymer, further corroborating the successful deposition of the polymer within the porous layer of the PSiO_2_ scaffolds.

A comparable Raman profile was observed for both NIP‐ and MIP‐coated PSiO_2_ scaffolds, likely because the propranolol functionalities may be masked by the poly(MAA‐co‐EGDMA) film, as previously reported for the imprinting of other drugs.^[^ ^65^ ^]^ Indeed, the vibrational modes could not emerge since the drug concentration was considerably lower than that of the polymer.

Propranolol sensing and rebinding experiments were further performed with the MIP‐based PSiO_2_ sensor (**Figure** **3**), using the NIP‐coated PSiO_2_ scaffold as a control, alongside with another PSiO_2_ scaffold coated with a polymer imprinted against atenolol, a structural analog of propranolol. Reflectance spectra were recorded after exposure to propranolol at concentrations in the range 5–1000 µm (Figure S12, Supporting Information) and EOT values retrieved for each tested concentration (Figure 3a). For the PSiO_2_ scaffolds functionalized with MIP for propranolol, the sensor response is significantly higher compared to NIP and MIP for atenolol, for all tested concentrations. Remarkably higher sensitivity is apparent for the MIP‐propanolol sensor from the linear portion of the curves, in the range from 5 to 100 µm (Figure 3b). The imprinting factor (IF), calculated as the sensitivity ratio (obtained from the slopes of the calibration curves) of MIP and NIP is ≈8.5, indicating a significant imprinting effect.^[^ ^66^ ^]^

Figure S13 (Supporting Information) reports the calibration curves recorded on MIP for propranolol and on MIP for atenolol, respectively. As expected, in the first case the responses to propranolol are significantly higher, with a 5.5‐fold higher sensitivity. Similarly, the atenolol MIP‐sensor exhibits a significantly higher sensitivity (about sevenfold) for atenolol. Remarkably, these results confirm the formation of specific binding sites determining the selective MIP‐template interaction and evidence at the same time the versatility of the proposed imprinting strategy which can be easily transferred to different targets. The limit of detection for propranolol (LoD)^[^ ^22^, ^36^ ^]^ estimated from the MIP calibration curve (Figure 3b) (calculated as LoD = 3.3d/m, where d is the residual standard deviation of the linear regression and m is the slope of the regression curve) is equal to 1.4 µm. The MIP‐based sensor exhibits satisfactory sample‐to‐sample reproducibility in the linear dynamic range with a relative standard deviation (RSD%) of 16% (*n* = 3, calculated considering the slope of the calibration curve). Remarkably, Figure 3c demonstrates that the sensor is reusable. The EOT value recorded on the same MIP‐based sensor after propranolol rebinding and subsequent washing confirms the ability of the sensor to be reused multiple times. A gentle washing procedure (MeOH/HAc, 9:1, v/v, for 10 min) is indeed sufficient to remove propanolol from the MIP, restornig the sensor for subsequent use.

**Figure 4:**
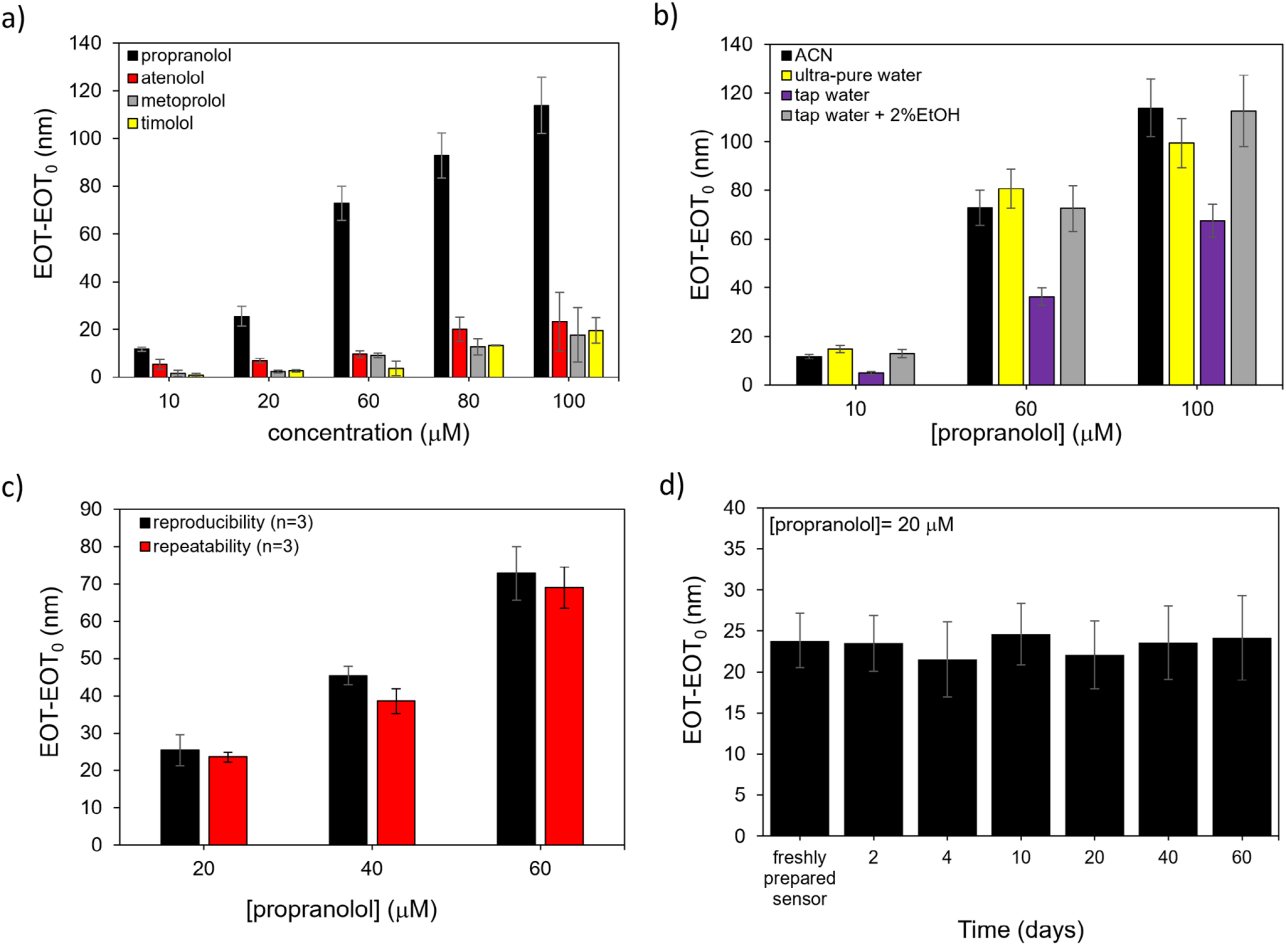
MIP‐PSiO_2_ sensor performance in propranolol optical detection. a) Selectivity results comparing the MIP sensor response (calculated as EOT‐EOT_0_; EOT_0_ is measured in blank solution and used as reference) to propranolol and to interfering compounds, namely, atenolol (AT), metoprolol (ME) and timolol (TI) at different concentrations (*n* = 3). b) MIP sensor response (calculated as EOT‐EOT_0_) in ultra‐pure and tap water (with or without 2% ethanol, v/v) spiked with propranolol at different concentrations. c) Comparison between MIP sensor response (calculated as EOT‐EOT_0_) recorded from three different samples (reproducibility, *n* = 3) and on three consecutive measurements on the same sensor (repeatability, *n* = 3). d) Long‐term monitoring of MIP sensor responses (EOT‐EOT_0_) over 60 days at a propranolol concentration of 20 µm.

To assess the ability of the MIP to selectively bind propranolol, the sensor was tested at increasing concentrations of interfering molecules that are structural and functional analogs of propranolol, namely, timolol (TI), atenolol (AT), and metoprolol (ME)^[^ ^67^, ^68^ ^]^ (**Figure** **4**a). The high MIP selectivity is evidenced by the higher sensor response to propranolol in comparison with the interfering molecules, although some interference is observed from AT and ME, particularly at high concentrations (100 µm), for which a significant response variation is recorded (RSD>30%).

The MIP‐based sensor then was used for detecting propranolol in ultra‐pure and tap water, compared to sensor responses in acetonitrile. As summarized in Figure 4b, sensor responses in ultra‐pure water closely resembled those in acetonitrile, while responses in tap water were lower. However, adding a small amount of ethanol (2%)^[^ ^67^ ^]^ to the tap water increased the response to a level comparable to that in acetonitrile. These tests highlight the sensor's potential for use in aqueous solutions with a simple and rapid sample pretreatment.

The reusability and stability of the MIP‐functionalized PSiO_2_ sensor were also evaluated (Figure 4c,d). The sensor exhibited satisfactory repeatability with an average RSD of 7% when tested multiple times. During a 60‐day storage period at room temperature under ambient conditions, the sensor showed an average variation of only 9% in its response. Regeneration of the sensor was achieved through a simple washing step with MeOH/HAc to remove propranolol from the binding sites. These findings demonstrate that the sensor can be stored and reused multiple times for at least two months.

The sensor's performance was compared with other MIP‐based sensors for propranolol reported in the literature (**Table** **1**).

**Table 1:** Comparison of the developed sensor with other MIP‐based optical sensors for propranolol from the literature.

MIP synthesis approach	Detection technique	Concentrations tested	Imprinting factor (IF)	Stability over time (days)	Real sample	Refs.
photopolymerization (by UV radiation)	SERS^a)^	115 µm	N.D.	N.D.	N.D.^b)^	[69]
photopolymerization (by UV radiation)	Raman Microspectroscopy	0.01 to 1000 µm	N.D.	N.D.	N.D.	[70]
precipitation polymerization	SERS	70 to 1000 µm	N.D.	N.D.	urine samples	[71]
precipitation polymerization	Fluorescence spectroscopy	246 fmol	4.5	N.D.	N.D.	[72]
precipitation polymerization	SERS	100 nm	4	N.D.	N.D.	[73]
precipitation polymerization	SERS	340 µm	N.D.	N.D.	N.D.	[74]
surface‐initiated thermal raft polymerization	UV–vis spectroscopy	3–200 µm	4.5	20 cycles (consecutively)	human plasma	[75]
micro‐emulsion polymerization	Fluorescence spectroscopy	0.8–65.0 nm	N.D.	N.D.	human blood	[76]
bulk thermal polymerization	Phosphorescence	1–2.5 µm	N.D.	N.D.	urine samples	[77]
photopolymerization (by UV radiation)	Fluorescence spectroscopy	4–155 µm	N.D.	`N.D.	N.D.	[78]
precipitation polymerization	Fluorescence spectroscopy	1 to 50 µM	7	N.D.	N.D.	[79]
photo‐iniferter polymerization (low‐energy visible light)	UV–vis spectroscopy	5–1000 µM	8.5	60	tap water	This work

^a)^ SERS: surface‐enhanced Raman scattering;

^b)^ N.D.: not declared.

Notable advantages of the optical MIP‐based sensor proposed in this study include its broad detection range, the capability to analyze real samples with minimal pre‐treatment (requiring only the addition of 2% ethanol), and long‐term usability of up to 60 days without the need for special maintenance. Furthermore, the MIP film was synthesized using low‐energy, environmentally friendly visible light. These attributes underscore the sensor's practicality and versatility for real‐world applications.

## 3 Conclusion

Herein, we have presented an innovative and versatile photo‐iniferter polymerization approach for direct MIP synthesis on nanostructured transducers. CDTPA was chosen as photo‐iniferter agent enabling polymerization under low‐intensity visible light and the highly conformable thin‐film MIP deposition was achieved through its covalent attachment to the nanotransducer surface.

As a proof of concept, thin MIP layers against propranolol as a model target were obtained on nanoporous silicon (PSiO_2_) interferometric transducers with a high aspect ratio (>100) and nanosized porous structure (≈50 nm). The resulting MIP showed remarkable performance in target detection in different media, including acetonitrile, ultra‐pure, and tap water, with high sensitivity (LOD of 1.4 µm), excellent specificity (imprinting factor (IF) equal to 8.5), good selectivity, and long‐term stability up to 60 days, with a response variability of only 9%. Moreover, the sensor exhibited satisfactory reproducibility (relative standard deviation (RSD%) of 16%), along with the ability to be easily regenerated after use. These findings highlight the sensor's potential for practical applications in the monitoring of drugs in different matrices. The successful application of the imprinting protocol to another template molecule, atenolol, reveals indeed the versatility of the proposed approach and its potential applications to different target molecules. Importantly, its easy transfer to any nanostructured transducers, taking advantage of the substrate‐independent deposition mechanism, represents an additional benefit, which allows further extending MIP applications in sensing and diagnostics.

## 4 Experimental Section

### Reagents

All chemicals were of analytical grade and used as received. The chemical reagents used include ethylene glycol dimethacrylate (EGDMA), 98%, methacrylic acid (MAA), ≥97.5%, acetonitrile, ≥99.9%, (3‐aminopropyl)triethoxysilane (APTES), 99%, aqueous hydrofluoric acid (HF, 48%), sodium hydroxide (NaOH, 98%), N,N'‐dicyclohexane carbodiimide (DCC), 99%, provided by Sigma‐Aldrich (Darmstadt, Germany); 4‐cyano‐4‐[(dodecylsulfanylthiocarbonyl) sulfanyl]pentanoic acid (CDTPA), ≥97%, was provided by TCI Chemicals (Tokyo, Japan). Diethyl ether (Et2O, >99%), methanol, ethanol, isopropanol, hydrogen peroxide (H_2_O_2_, 30%), and acetic acid (HAc) of analytical grade were purchased from Carlo Erba (Milan, Italy). Propranolol hydrochloride, 99%, atenolol, ≥98%, timolol maleate salt, ≥98% and metoprolol tartrate salt were provided by Thermo Fisher Scientific (Geel, Belgium).

High‐power, multi‐chip LED525‐66‐60 was provided by Roithner LaserTechnik (Vienna, Austria). This light source displays a maximum peak at 523 nm, with an FWHM of 30.5 nm, measured with a UV−vis spectrometer (SM242 SP) provided by Spectral products.

Silicon boron‐doped wafers (p++ type) with a resistivity of 0.8–1.2 mΩ × cm, orientation <100>, were purchased from Siltronix Silicon Technologies (France).

APTES solutions were prepared in toluene (2%, v/v), immediately before the use. CDTPA (5 mm) solutions containing DCC (5 mm), were prepared in DMF and left under stirring (15 rpm) using a rotating tube mixer for an overnight step (16 hs). A stock solution of propranolol (1 mm) was freshly prepared in acetonitrile before use. From this, propranolol standard solutions at different concentrations (from 5 to 1000 µm) were obtained. Standard solutions of atenolol, timolol, and metoprolol were obtained similarly. Alternatively, propranolol standard solutions were also prepared in ultra‐pure and tap water (5–100 µm).

### Porous Silicon (PSi) Substrates Preparation and Oxidation

PSi samples were prepared by a method already known in the literature.^[^ ^80^ ^]^ An electrochemical etching of silicon wafer (15 × 15 mm) was performed using a solution of hydrofluoric acid (48%) and ethanol (3:1, v/v). A two‐electrodes Teflon cell with a platinum wire cathode and an aluminum flat anode was employed to electrochemically etch silicon samples over a circular area of 0.567 cm^2^ by using a Keithley 2602A SourceMeter, setting current density and measuring the voltage. A first PSi sacrificial layer was etched at 600 mA cm^−2^ for 10 s and dissolved by alkaline dissolution with a solution of NaOH(1 m) and EtOH (9:1, v/v). The silicon samples were rinsed with ultra‐pure water, and ethanol and then dried under a gentle nitrogen flow. The PSi sensing layer (i.e., the PSi interferometer) was then etched at 600 mA cm^−2^ for 25 s on the so‐processed silicon samples, rinsed with isopropanol and diethylether, and gently dried under a nitrogen flow to achieve a crack‐free PSi layer. Thermal oxidation of the PSi interferometer was performed in muffle (Nabertherm, Lilienthal, Germany) at 1000 °C for 10 min (ramp‐up/ramp‐down 15 °C min^−1^) to obtain PSiO_2_ samples.

### Characterization of PSiO_2_ Interferometers and FFT Reflectance Spectroscopy

Reflectance spectra of the PSiO_2_ interferometers were acquired in the air in the wavelength range (400−1000 nm) using an optical setup consisting of a UV−vis spectrometer (SM242 SP) provided by Spectral products, a bifurcated fiber‐optic probe (QR200−7‐VIS‐BX) and a lamp source (HL‐2000) provided by Ocean Optics (USA). Light from the halogen lamp source is fed orthogonally onto the PSiO_2_ surface and the light reflected from the PSiO_2_ layer is collected into a UV−vis spectrometer by the fiber‐optic probe. Acquisition parameters for reflection spectra were: integration time 50 ms, average scan number 15, boxcar width 5, with the spectrometer working in photon counts mode. The porosity of as‐prepared PSiO_2_ interferometers was estimated by best‐fitting the reflectance spectra of PSi layers acquired before oxidation.^[^ ^80^ ^]^ PSiO_2_ prepared from p‐type silicon wafer^[^ ^81^, ^82^ ^]^ exhibits well‐defined Fabry–Perot fringes in the reflectivity spectrum whose position is governed by the relationship:

(2) mλ=2nL

where m is the spectral order of the optical fringe, λ the wavelength at which each interference maximum appears, n is the refractive index of the film, and L is its thickness.

FFT of the reflectance spectra of PSiO_2_ interferometers was performed to calculate the EOT values, namely, 2 nL, where *n* = effective refractive index and L = thickness of the PSi layer, using a home‐made software (MatLab, MathWorks, USA). The wavelength axis of the reflectance spectrum was first inverted (*x* axis changed from wavelength to 1/wavelength) to obtain a wavenumber axis. A cubic‐spline interpolation of reflectance data was then carried out to obtain a dataset (reflection, wavenumber) spaced evenly (sample‐to‐sample distance 8.57 × 10^−7^ nm^−1^). A Hanning window was applied to the reflectance spectrum, which was zero padded to 224. Eventually, application of the FFT algorithm to the zero‐padded reflectance spectrum yielded the Fourier transform amplitude and phase (*y* axis in the Fourier transform domain) as a function of 1/wavenumber (*x* axis in the Fourier transform domain), with a spatial resolution of ≈0.07 nm. The EOT value is obtained as the value of the 1/ wavenumber axis (*x* axis) in the Fourier transform domain for which the main peak in the Fourier transform amplitude (*y* axis) occurs.^[^ ^80^ ^]^

### Polymer Deposition by Photo‐Iniferter Polymerization on Flat Silicon and Contact Angle Measurements

Slides of silicon wafers (3 mm x 10 mm) were first thermally oxidized (750 °C for 1 h in a muffle), to obtain a thin layer of oxide (SiO_2_) on their surface.^[^ ^83^ ^]^ Subsequently, they were used during polymer deposition tests. The experimental conditions used are described in more detail in the section “PSiO_2_ functionalization with molecularly imprinted polymer films”. Different deposition times (from 10 min to 8 h) were explored and the surface modifications of the silicon slides were monitored by contact angle (CA) measurements. CA was recorded after each functionalization step of flat silicon slides. The experimental apparatus used for CA measurements consists of a chamber containing a holder sample (a plate) located between a light source and a high‐resolution camera.^[^ ^84^ ^]^ The sample is placed on the plate and a small water droplet (5 µL) is dropped on its surface. The camera presented a high‐quality image of the water drop and the exact contact angle was measured using ImageJ software (version 1.54d, Wayne Rasband, National Institutes of Health, United States). For each sample, several pictures (*n* = 5) were recorded, on different positions (*n* = 3).^[^ ^84^ ^]^

### PSiO_2_ Functionalization with Molecularly Imprinted Polymer Films

PSiO_2_ scaffolds were first cleaned by treatment with piranha solution (H_2_SO_4_:H_2_O_2_, 3:1, v/v)^[^ ^85^ ^]^ for 10 min at 40 °C. Later, they were immersed in a solution of APTES prepared in toluene (2%, v/v) for 30 min at 55 °C,^[^ ^85^, ^86^, ^87^ ^]^ then washed with water for 5 min, rinsed with ethanol and gently dried with a nitrogen flow. Next, the silanized samples were immersed in CDTPA (5mM) solutions containing DCC (5 mM), prepared in THF, for an overnight step (16 h) to graft the iniferter to the surface of PSiO_2_ scaffolds by a coupling reaction. On CDTPA‐grafted PSiO_2_ scaffolds propranolol imprinted films (MIP_Prop_) were synthesized as follows. Propranolol (155 mg, 0.59 mmol), MAA (411.6 µL, 4.73 mmol), and EGDMA (4.55 mL, 23.67 mmol), were dissolved in anhydrous acetonitrile (for a total volume of 10 mL) to obtain the pre‐polymerization mixture. CDTPA‐grafted PSiO_2_ samples were vertically placed in a 10‐mL glass vial, which was filled with the pre‐polymerization solution and closed with a silicone septum. Later, the solution was nitrogen‐purged on ice (30 min), and then CDTPA‐grafted PSiO_2_ scaffolds were placed in front of a LED irradiating green visible light, to trigger the polymerization. The distance to the LED was set to measure an average power of 1.5 mW cm^−2^ with a Coherent PS19Q sensor. The optimal MIP deposition time corresponds to 5 h although different polymerization times (3, 4, 5, and 8 h) have been explored. After the deposition of the MIPs on PSiO_2_ scaffolds, the samples were repeatedly rinse with water and then methanol, to remove any adsorbed polymerization solution and finally dried with a gentle nitrogen flow. To obtain the imprinted cavities on the deposited MIP, a classical washing procedure,^[^ ^88^, ^89^ ^]^ was performed. In brief, MIP‐grafted PSiO_2_ scaffolds were placed in MeOH/HAc solutions (9:1, v/v), under stirring (200 rpm) for 1 h at room temperature, rinsed with water, and methanol, and then dried. The washing step was repeated, if necessary until a stable reflectance spectrum of the MIP‐coated PSiO_2_ scaffolds was achieved. Alternatively, molecularly imprinted polymer films against atenolol were also obtained similarly but replacing propranolol with atenolol in the polymerization solutions.

As a reference, not‐imprinted polymers (NIPs) were synthesized as previously described but using a polymerization solution without target molecules. Likewise, with MIPs, NIPs were subjected to washing procedures.

### Synthesis of Bulk Polymers by Photo‐Iniferter Polymerization

To obtain bulk polymers, a polymerization solution (10 mL) prepared in anhydrous acetonitrile and containing propranolol (155.5 mg, 0.59 mmol), MAA (411.6 µL, 4.73 mmol), EGDMA (4.55 mL, 23.67 mmol), and CDTPA (1% of the overall concentration of monomer and crosslinker), was used. The solution was nitrogen‐purged on ice and then placed in front of an LED irradiating green visible light, to trigger the polymerization. Again, the distance to the LED was set to measure an average power of 1.5 mW cm^−2^. A polymer deposition time of 5 h was adopted. After the polymerization, a bulk polymer was obtained and stored up to its characterization by XPS.

### Morphological Characterization of PSi and PSiO_2_ Scaffolds Before and After Polymer Deposition

Top‐view morphological characterization of PSi and PSiO_2_ scaffolds before and after polymer deposition was carried out using a scanning electron microscope (FEG‐SEM, Zeiss SUPRA) with a 10 kV acceleration voltage at various magnifications. Distribution of the pore diameters was obtained from the analysis of top‐view SEM images using the “Equivalent Disc Radius” function in the Grain Distribution tool of the Gwyddion software.

### XPS Characterization

XPS spectra before and after each functionalization step of PSiO_2_ samples were recorded with an AXIS ULTRA DLD (Kratos Analytical) photoelectron spectrometer using a monochromatic source (1486.6 eV) at 150 W (10 kV, 15 mA) and 5.3 × 10^−9^ torr as base pressure in the analysis chamber. The survey scan spectra were recorded with a pass energy of 160 and a 1 eV step, while the high‐resolution spectra were obtained with a pass energy of 20 and a 0.1 eV step. In both cases, the analysis area was ≈0.3 mm × 0.7 mm. The processing of the XPS spectra was performed using CasaXPS release 2.3.16 software, where the binding energy (BE) scale was referenced to the Au 4f7/2 peak at 84.0 eV. During the analysis of the high‐resolution spectra, all the peaks were fitted with Shirley background and GL(30) lineshape (Gaussian 70%, Lorentzian 30%). For quantitative analysis, the relative sensitivity factors from the CasaXPS library were used for the signal areas. The surface charging was rectified by considering adventitious C 1s (binding energy, BE = 285 eV).

### Binding Kinetics and Rebinding Tests

The effect of contact time (or incubation time) between MIP and target on the sensor response was evaluated in a time range of 10–60 min. Freshly prepared propranolol (50 µm) standard solutions were placed (50 µL) on a PSiO_2_ scaffold functionalized with the propranolol imprinted polymer, with the interferometer placed in a closed chamber (a 3‐cm glass petri dish) and there left for different incubation times. After incubation, the sensors were washed with water (3 min, under stirring), rinsed with ethanol, and then dried under a nitrogen flow. The reflectance spectra were recorded before and after the exposure of the sensor with the propranolol solutions. Before a new experiment, MIPs were restored by a washing procedure (10 min, under stirring) in MeOH/HAc (9:1, v/v).

During the rebinding tests, MIP‐functionalized PSiO_2_ samples were exposed to increasing concentrations of target standard solutions prepared in acetonitrile (5–1000 µm). The MIP‐based sensors were placed in a closed chamber to avoid the evaporation of the standard solutions. The reflectance spectra were recorded before and after each propranolol concentration tested. All experiments were carried out in triplicate (*n* = 3). Alternatively, atenolol detection tests were also performed using atenolol imprinted polymers, under the same conditions. MIP and NIP sensitivity were estimated from the slope of the calibration curves.

MIP‐functionalized PSiO_2_ samples were also used for propranolol detection tests in ultra‐pure and tap water to evaluate the potential use of the sensor in real applications. Propranolol stock solution was spiked in ultra‐pure and tap water and the rebinding experiments were performed under the same conditions used for the calibration.

### Selectivity, Repeatability, and Stability Tests

MIP selectivity was evaluated by testing MIP‐based sensors with solutions containing different interfering molecules such as atenolol (AT), timolol (TI), and metoprolol (ME) at different concentrations (10–100 µm). Freshly prepared solutions were prepared immediately before the experiments.

Repeatability was assessed by testing the MIP‐PSiO_2_ sensor for propranolol detection in three consecutive experiments with the same sensor. Again, to regenerate the MIP sufficient a treatment with MeOH/HAc (9:1, v/v) for 10 min.

The time stability of the MIP‐based sensor for propranolol detection was evaluated by monitoring the sensor response at 20 µm for different time intervals up to 60 days. The sensor was stored in the air without any particular care and washed with MeOH/HAc (9:1, v/v) for 10 min, before its use.

## Conflict of Interest

The authors declare no conflict of interest.

## Supporting information



Supporting Information

## Data Availability

The data that support the findings of this study are available in the supplementary material of this article.
