# Supplementary material for: Visible‐Light Photo‐Iniferter Polymerization of Molecularly Imprinted Polymers for Direct Integration with Nanotransducers
Source: Small Methods. 2025 Jan 24;9(4):2401315. doi: 10.1002/smtd.202401315 (PMC12020366; doi:10.1002/smtd.202401315)
Supplement: Supplementary file 1 — Supporting Information [file SMTD-9-2401315-s001.docx]

Supporting Information

**Visible-light photo-iniferter polymerization of molecularly imprinted polymers for direct integration with nanotrasducers**

Tiziano Di Giulio^1^, Ibrar Muhammad Asif^1^, Martina Corsi^2^, Giuseppe Egidio De Benedetto^3^ Cosimino Malitesta^1^, Karsten Haupt^4,5^, Giuseppe Barillaro^2,*^, Carlo Gonzato^4,*^, Elisabetta Mazzotta^1,*^

^1^Laboratory of Analytical Chemistry, Department of Biological and Environmental Sciences and Technologies (Di.S.Te.B.A.), University of Salento, via Monteroni, 73100 Lecce (Italy)

^2^Information Engineering Department, University of Pisa, via G. Caruso 16, 56122 Pisa (Italy)

^3^Laboratory of Analytical Mass Spectrometry, Cultural Heritage Department, University of Salento, Via Monteroni, 73100 Lecce (Italy)

^4^CNRS Enzyme and Cell Engineering Laboratory, Université de Technologie de Compiègne, Rue du Docteur Schweitzer, CS 60319, Compiègne 60203 (France)

^5^Institut Universitaire de France

[*giuseppe.barillaro@unipi.it*](mailto:giuseppe.barillaro@unipi.it)*; carlo.gonzato@utc.fr;* [*elisabetta.mazzotta@unisalento.it*](mailto:elisabetta.mazzotta@unisalento.it)*.*


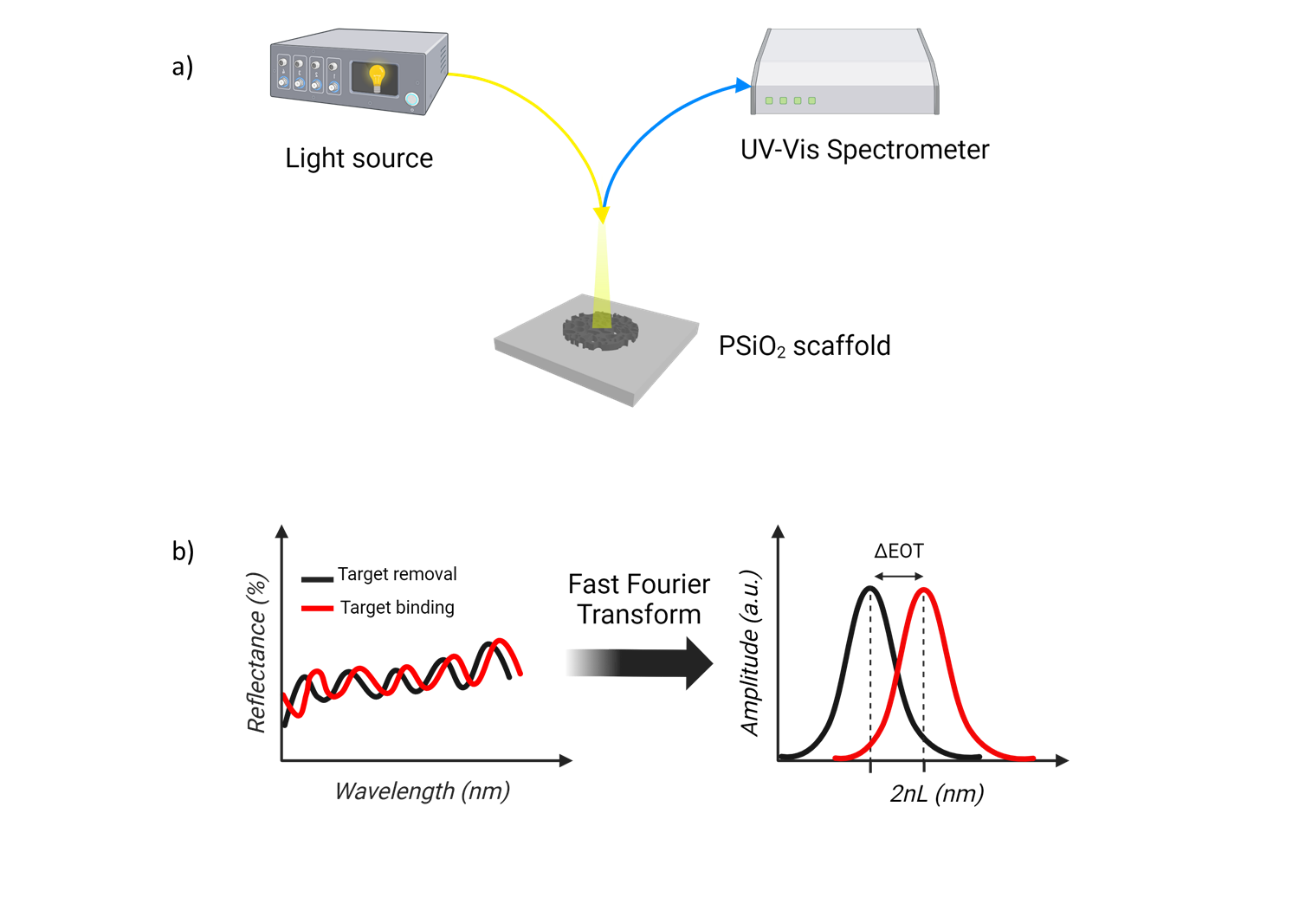


**Figure S1.** a) Schematic of the setup used for reflectance measurements, featuring a halogen lamp as the light source and a UV-Vis spectrometer. b) Illustrative graph of reflectance spectra used to calculate EOT values, which serve as a parameter for the functionalization steps and for monitoring binding and removal events in the MIP sensor. Created in https://BioRender.com


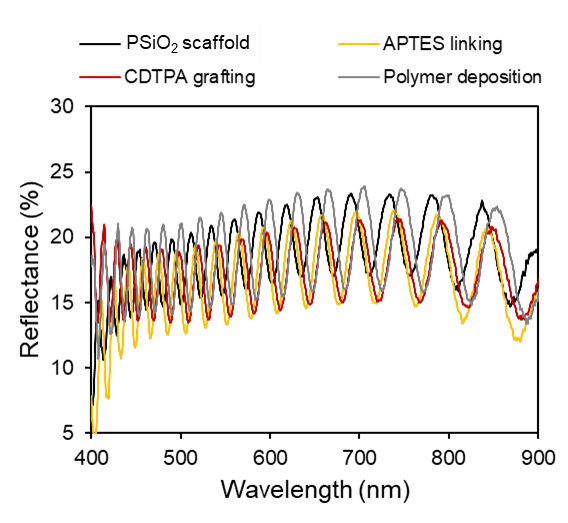


**Figure S2**. **Optical characterization of PSiO_2_ scaffold during photo-iniferter polymerization for polymer deposition.** Reflectance spectra recorded in air on a PSiO_2_ scaffold before and after each functionalization step up to the polymer deposition.


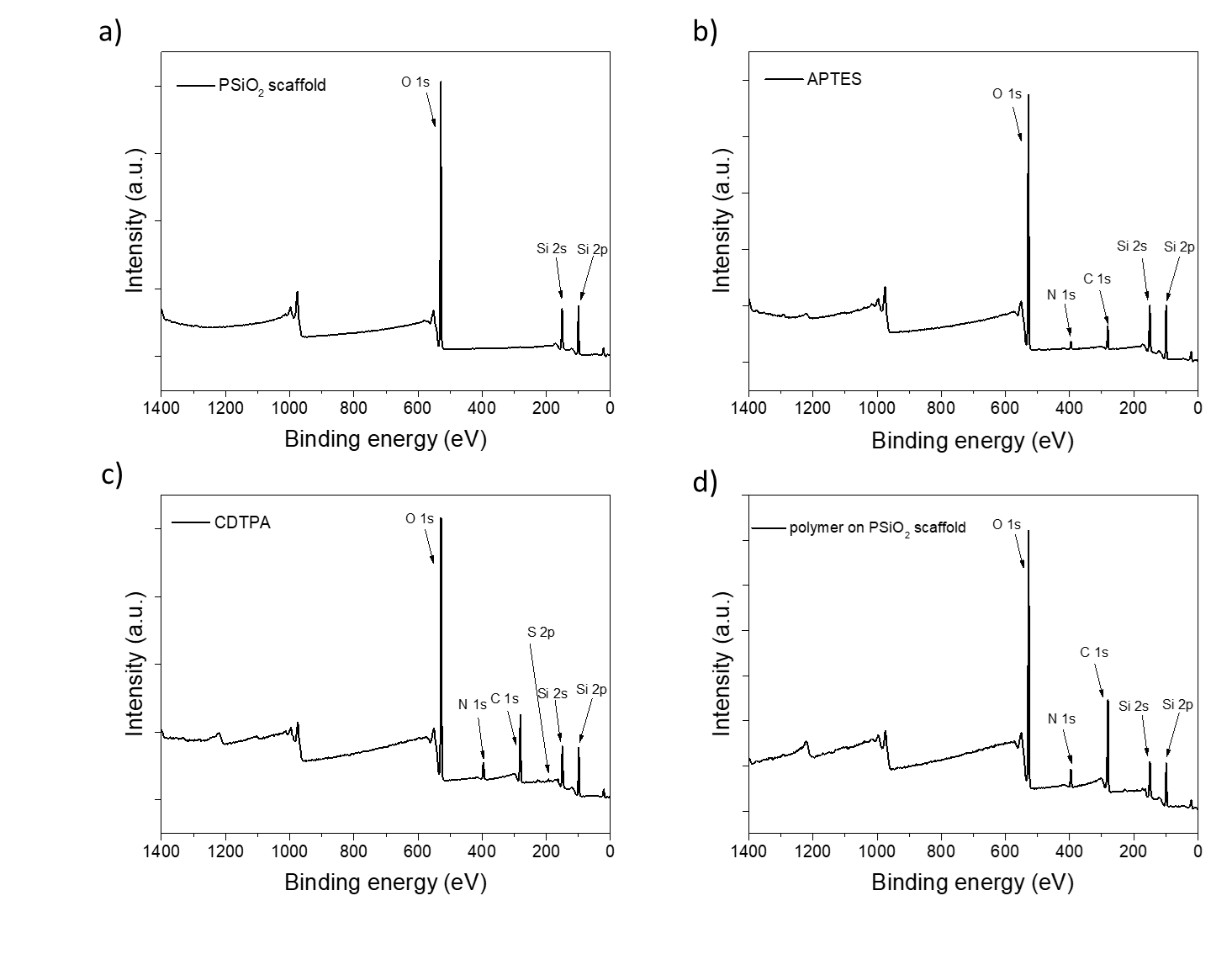


**Figure S3**. **X-ray photoelectron spectroscopy characterization of PSiO_2_ scaffolds upon photo-iniferter polymerization.** Survey scan recorded on a) bare PSiO_2_ scaffold; b) after APTES silanization; c) after CDTPA grafting and d) after the polymer deposition (5 hours polymerization).


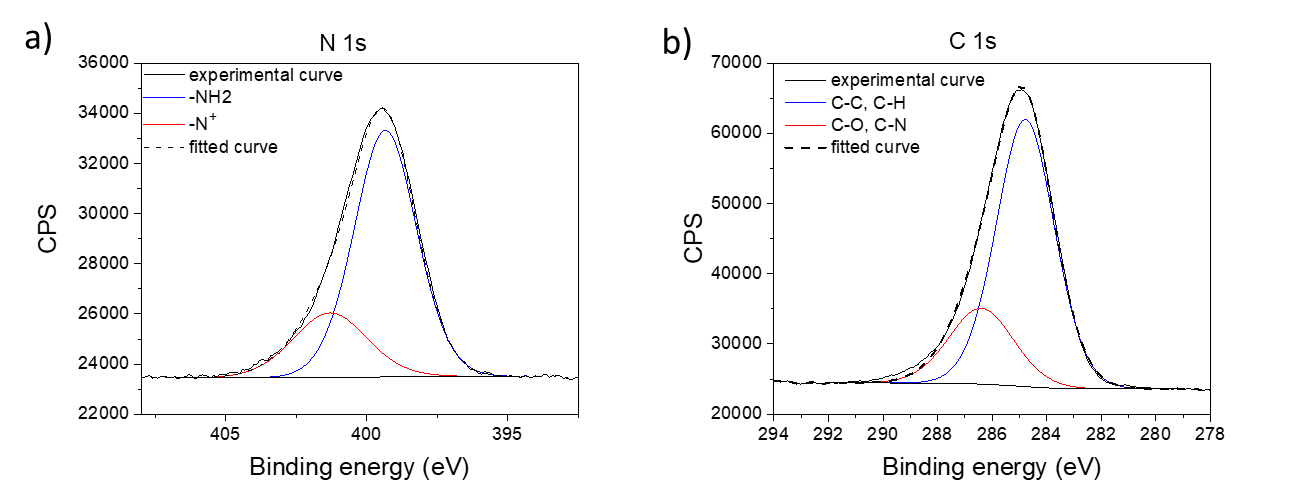


**Figure S4**. **X-ray photoelectron spectroscopy characterization of PSiO_2_ silanized with APTES.** N1s (a) and C 1s (b) signals recorded after PSiO_2_ silanization with APTES.


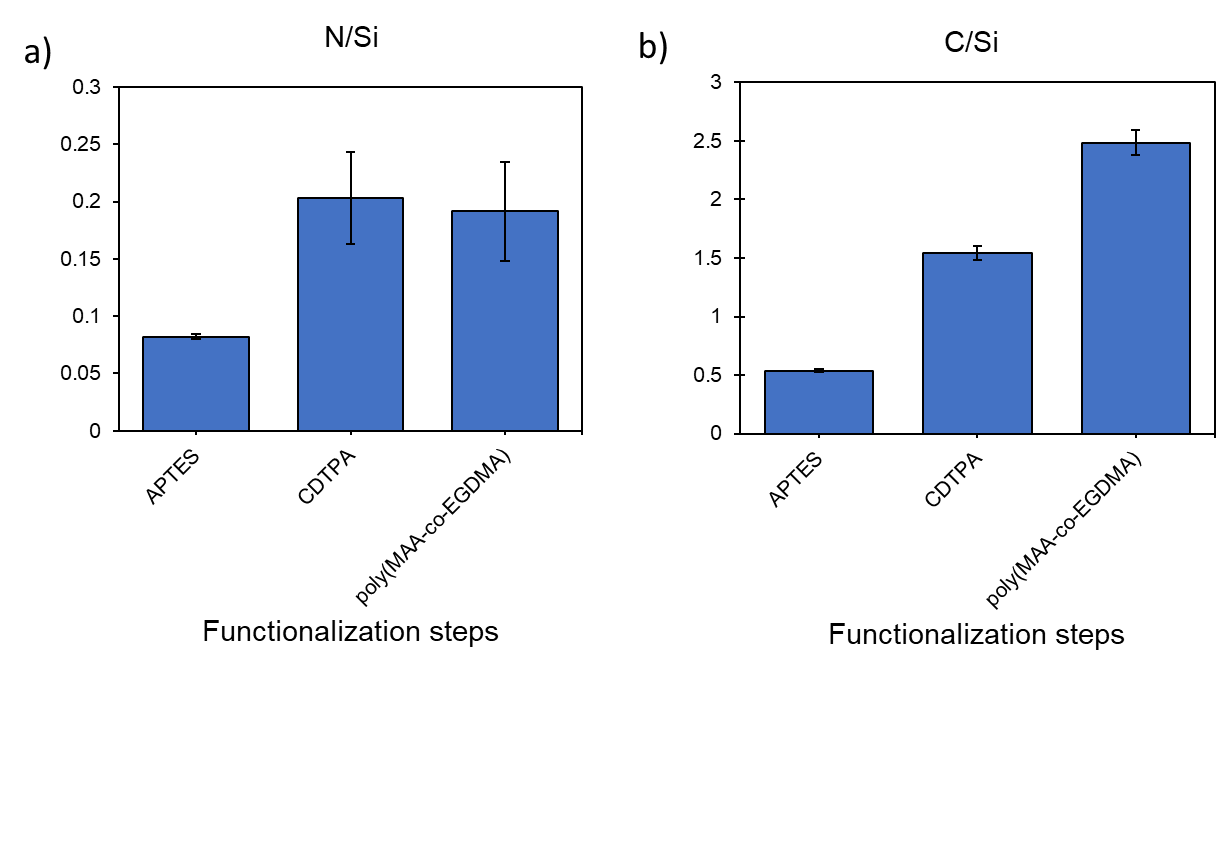


**Figure S5. Relative atomic ratio calculated from XPS data.** N/Si (a) and C/Si (b) ratio calculated for each functionalization step of PSiO_2_ samples for poly(MAA-co-EGDMA) deposition.


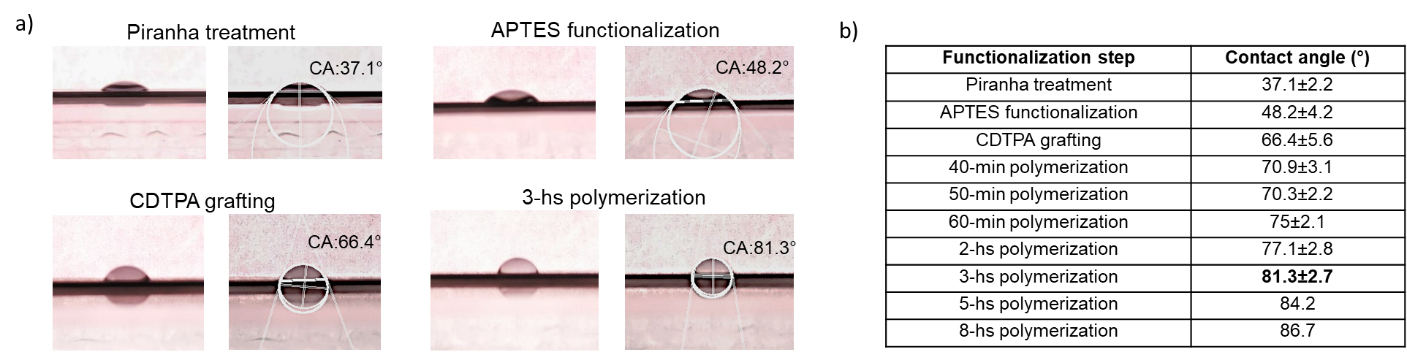


**Figure S6**. **Contact angle measurements on Si wafer slides.** a) contact angle recorded during the functionalized steps up to the polymer deposition. b) average contact angle values recorded for each functionalization step and after photo-iniferter polymerizations performed at different times (n=3). Data are presented as mean (± s.d).

**Figure S7**. **Optical characterization of PSiO_2_ scaffold after polymer deposition for different polymerization times.** Reflectance spectra recorded in air on a PSiO_2_ scaffold before and after photo-iniferter polymerization for different times.


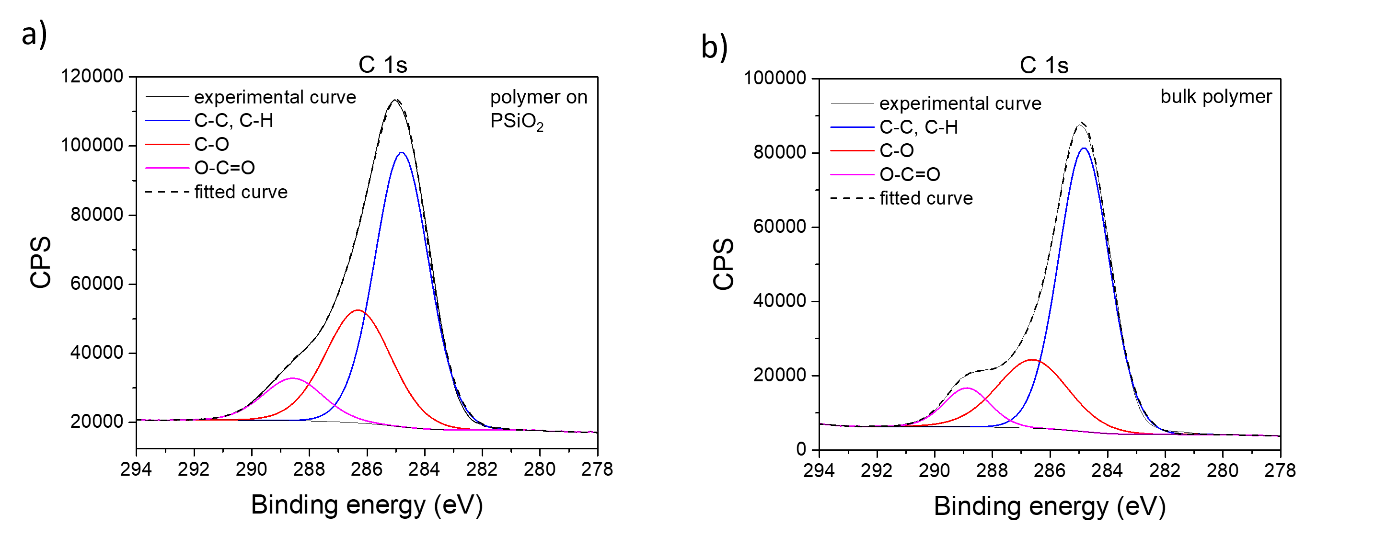


**Figure S8**. **X-ray photoelectron spectroscopy characterization of the polymer obtained by photo-iniferter polymerization.** High-resolution C1s signal recorded after polymerization of poly(MAA-co-EDGMA) on PSiO_2_ (a) and in solution as a bulk polymer (b).


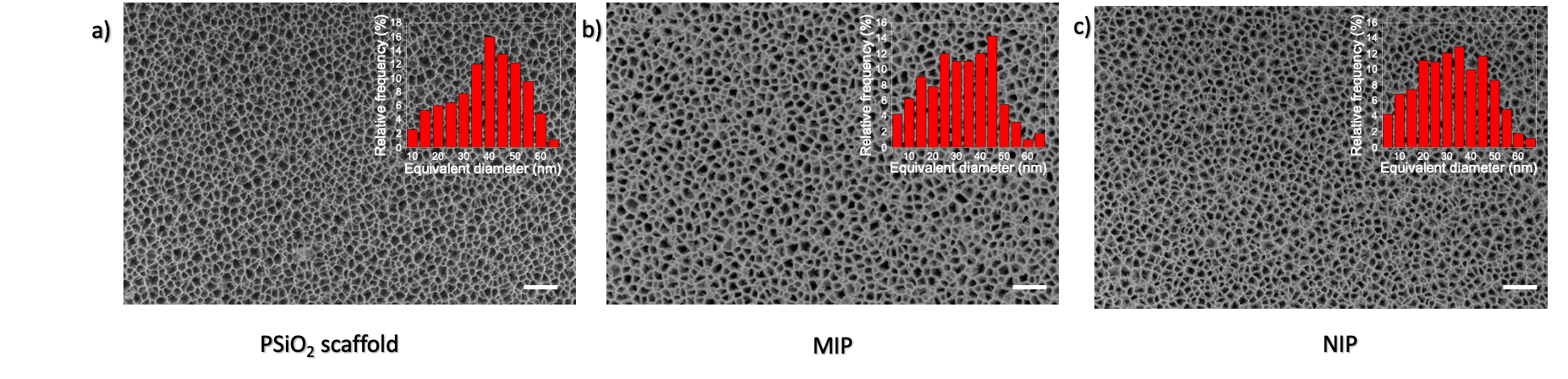


Figure S9. Top-view SEM images and characterization of PSiO2 scaffolds after deposition of MIP and NIP for 5 hours. Distribution of the pore diameters was obtained from the analysis of top-view SEM images using the “Equivalent Disc Radius” function in the Grain Distribution tool of the Gwyddion software. Scalebar: 200 nm.


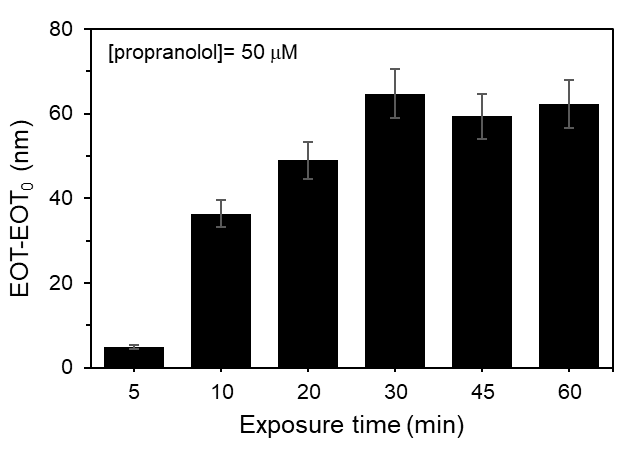


**Figure S10**. **MIP kinetic studies.** Sensor response (EOT-EOT_0_ versus propranolol concentration) recorded after the incubation of MIP-functionalized PSiO_2_ scaffolds for different contact times, namely 5, 10, 20, 30, 45 and 60 minutes; EOT_0_ is the signal recorded for the blank solution and used as reference. Propranolol concentration: 0.05 mM. MIP polymerization time: 5 hours.

**Figure S11**: **MIP characterization by Raman spectroscopy**. Raman spectra of bare PSiO_2_ (dark line), NIP-coated (blue line) and MIP-coated (red line) PSiO_2_ scaffolds recorded from 100 to 1800 cm-1. As control, a Raman spectrum of a bulk polymer was also recorded (green line) The bulk polymer was obtained using the same method but with the photo-iniferter (at 1% of the total concentration of monomer and crosslinker) in the polymerization mixture.


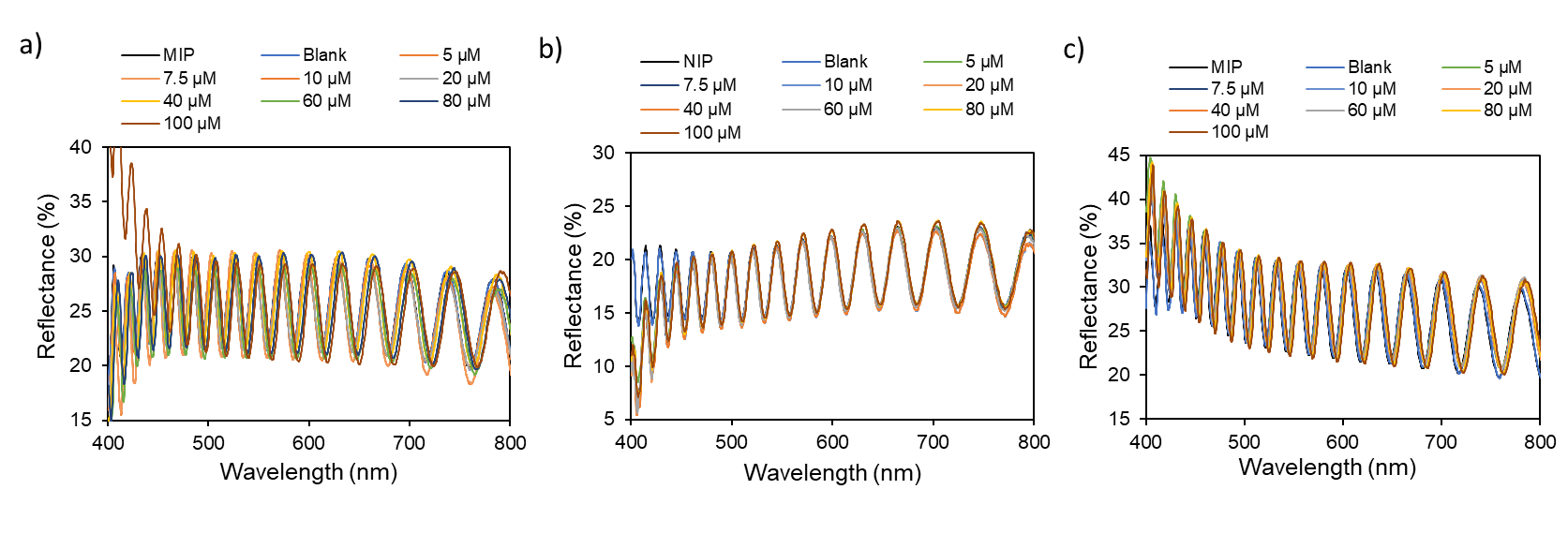


**Figure S12. Detection tests using MIPs- and NIPs-based sensors.** Reflectance spectra recorded in air on a) PSiO_2_ scaffold functionalized with MIP for propranolol after exposure to different concentrations of propranolol. b) NIP-coated PSiO_2_ sample after exposure to different concentrations of propranolol. c) PSiO_2_ scaffold functionalized with MIP for atenolol after exposure to different concentrations of atenolol.


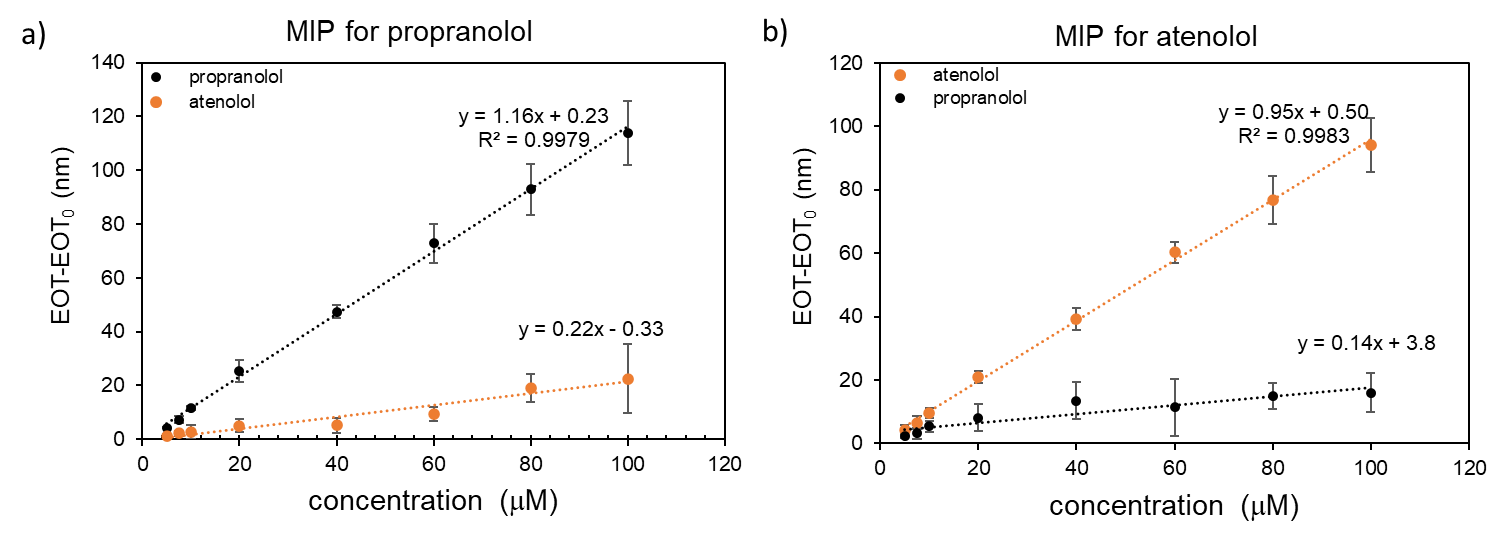


**Figure S13**. **MIP-based sensor performance in propranolol and atenolol detection.** a) Calibration curves (EOT-EOT_0_) recorded using propranolol-imprinted polymers for propranolol (black dots) and atenolol (orange dots) detection. b) Calibration curves (EOT-EOT_0_) recorded using atenolol-imprinted polymers for propranolol (black dots) and atenolol (orange dots) detection
